# Imaging Approaches and the Quantitative Analysis of Heart Development

**DOI:** 10.3390/jcdd10040145

**Published:** 2023-03-29

**Authors:** Morena Raiola, Miquel Sendra, Miguel Torres

**Affiliations:** 1 Cardiovascular Regeneration Program, Centro Nacional de Investigaciones Cardiovasculares (CNIC), 28029 Madrid, Spain; morena.raiola@cnic.es (M.R.); msendra@cnic.es (M.S.); 2 Departamento de Ingeniería Biomedica, ETSI de Telecomunicaciones, Universidad Politécnica de Madrid, 28040 Madrid, Spain; 3 Centro de Investigación Biomédica en Red de Enfermedades Cardiovasculares (CIBERCV), 28029 Madrid, Spain

**Keywords:** heart morphogenesis, imaging techniques, scanning laser, stereology, tomography, quantitative analysis

## Abstract

Heart morphogenesis is a complex and dynamic process that has captivated researchers for almost a century. This process involves three main stages, during which the heart undergoes growth and folding on itself to form its common chambered shape. However, imaging heart development presents significant challenges due to the rapid and dynamic changes in heart morphology. Researchers have used different model organisms and developed various imaging techniques to obtain high-resolution images of heart development. Advanced imaging techniques have allowed the integration of multiscale live imaging approaches with genetic labeling, enabling the quantitative analysis of cardiac morphogenesis. Here, we discuss the various imaging techniques used to obtain high-resolution images of whole-heart development. We also review the mathematical approaches used to quantify cardiac morphogenesis from 3D and 3D+time images and to model its dynamics at the tissue and cellular levels.

## 1. Introduction

### 1.1. Heart Morphogenesis

The heart is the first functional organ formed in mammalian embryogenesis. After the onset of gastrulation, a primitive heart is assembled and begins to pump nutrients throughout the embryo while it continues to grow. The heart’s ability to simultaneously form and function has captivated researchers for almost a century, but imaging this complex morphogenetic processes remains a challenge for the field [1,2,3,4].

**Figure 1:**
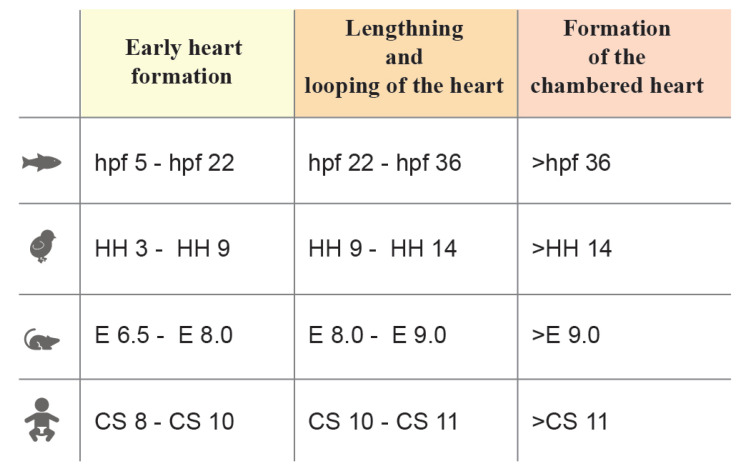
Stages of heart morphogenesis in vertebrate model organisms (zebrafish, chick, mouse, and human). Stage windows are reported in hours post fertilization (**hpf**) for zebrafish, Hamburger-Hamilton (**HH**) for chick embryo, embryonic day (**E**) for mouse embryo and Carnegie stages (**CS**) for human.

Heart morphogenesis occurs following similar phases in different species [5,6,7]. Heart development can be grossly divided into three stages: 1. Primitive heart tube morphogenesis, 2. Looping, lengthening, and chamber formation and 3. Chamber maturation, trabeculation, and valve formation (see Figure 1). In the first stage, cardiac progenitors in the epiblast ingress through the primitive streak into the mesodermal layer and then migrate to the anterior part of the embryo and differentiate [8]. These progenitors, known as the first heart field (FHF), are arranged in a horseshoe-shaped primordium in the mouse and two cardiac primordia in human, avian and zebrafish embryos [9,10,11,12], eventually forming a rudimentary beating hemi-tube called the primitive heart tube. In the second stage, the second heart field (SHF) progenitors, which are placed posteromedially and immediately adjacent to the cardiac crescent in the splanchnopleura, begin to differentiate and lengthen the primitive heart tube at the arterial and venous poles [2,13,14]. As the beating becomes more consistent, the heart undergoes looping, extensive growth, and morphological modifications that lead to the formation of a partially septated four-chambered heart, equipped with a set of immature valves and trabeculae [4,15]. In the final stage, the subdivision of the outflow tract and the complete interventricular and atrial septation define the morphology of the mature heart [16].

### 1.2. Challenges in Imaging Heart Development

These rapid and complex changes in heart morphology make imaging during development a challenging task. In its initial stages, the heart tube is relatively translucent and exposed to the outside of the embryo. However, cell progenitors migrate and differentiate rapidly, causing sudden morphological changes that can only be understood by imaging fixed and live specimens [2,17]. At later stages, such live imaging techniques cannot resolve the thicker and more complex structures of the heart, which become three-dimensional and internalized in the embryo. Instead, the dissection, fixation, and clarification of the sample allow us to obtain detailed 3D images of the heart [18,19,20].

The way the heart develops poses some challenges for their imaging and quantification in live embryos, but researchers have taken advantage of the features of different vertebrate model organisms. For instance, the zebrafish’s translucent embryo and rapid development make it the most suitable model for obtaining high-resolution images in live embryos [21]. The chicken embryo, due to its accessibility to physical manipulation and culture, offers opportunities for imaging migration and differentiation of the heart field [22]. However, efficient methods for genetic manipulation of the chicken embryo are still limited [23]. The mouse embryo, with a recent boost of advances in imaging and culture methods [2,17,24,25,26], is now also a promising model for imaging the early stages of cardiac morphogenesis. Although some advances have been made to image looping and trabeculation stages in the mouse embryo [15,27], it is still challenging to obtain high-resolution images when the heart internalizes deep within the embryo; however, this is a limitation in all models.

### 1.3. Quantitative Approaches towards Understanding Heart Morphogenesis

Advances in imaging technology improved our ability to study heart development [2,27]. The integration of multi-scale and live imaging approaches with genetic labeling has unlocked new insights into the molecular mechanisms shaping the heart both at the tissue and cellular levels [17,28,29,30]. These types of data required advanced image processing methods to extract relevant biological information. Over time, the field of computer graphics and machine learning has introduced powerful tools to characterize the processes that drive cardiac morphogenesis in a quantitative manner.

The three-dimensional nature of heart development makes segmentation and 3D reconstruction essential tools to interpret whole-mount images. For the simple geometries of single cells, such as nuclei or cell membranes, 3D segmentation allows one to extract geometric features which are relevant to identify the cell state. These include volume, polarity, orientation, and anisotropy, among others. Several approaches have been proposed for automated membrane/nuclear segmentation. Popular built-in models for this type of segmentation include Ilastik [31], Weka [32], Cellpose [33], MorphoLibJ [34], and StarDist [35], among others. More complex 3D geometries of tissues composed of multiple cell layers, such as the heart tube or trabeculae, may still require specific markers or manual steps to segment different tissue layers. Once the segmentation is reconstructed in 3D, it can be discretized in space into a polygonal mesh, creating an object that can be easily visualized, measured, and manipulated in several open-source software such as Meshlab [36].

In this review, we first discuss and compare the applications of different imaging methods used for whole-heart imaging, including scanning-laser fluorescence microscopy, such as confocal, two-photon, and light-sheet imaging, stereological approaches, such as serial histology or high-resolution episcopic imaging (HREM), and tomographic approaches, such as micro-computed tomography (micro-CT), optical coherence tomography (OCT), and optical projection tomography (OPT) (compared in Table 1). Then, we review quantification strategies to extract biologically meaningful features from these images. We focus on the mathematical approaches implemented to quantify cardiac morphogenesis at the tissue and cellular scales from 3D (static) and 3D+time (live) images summarized in Table 2, Table 3 and Table 4.

## 2. An Overview of Imaging Methods

### 2.1. Fluorescence Microscopy

Fluorescence microscopes have undergone significant improvements since their invention in the early 20th century, including advances in optics, excitation sources and detection systems. Currently, widefield epifluorescence microscopes remain as versatile tools for various applications, including visualizing whole tissue mounts and evaluating expression rates of fluorescent tags [37,38].

Confocal microscopy, invented in the 1950s [39], represented a further advance in fluorescence microscopy, offering higher spatial resolution and the ability to visualize subcellular details [40]. This technology uses lasers to scan a sample point by point, while a pinhole blocks unfocused light from the imaging plane, enabling the detection of optical sections in thick samples [41]. This approach forms the basis of other laser scanning microscopes tailored to specific applications [42,43].

An example of the use of confocal microscopy in heart development are the recent efforts to map single-cell RNA sequencing (scRNAseq) clusters onto developing tissues using fluorescent in situ hybridization (FISH) [29,30]. The authors first performed scRNAseq to identify putative cell populations within the developing heart and then used FISH to visualize the expression of genes associated with each population. These studies show the power of combining scRNAseq with FISH and confocal imaging to study gene expression in complex tissues.

In addition to identifying the expression patterns of specific RNAs and proteins in whole embryos, confocal microscopy can also be used to obtain high-resolution images of samples up to 700 μ m thick, when combined with genetic fluorescent reporters and clarification methods [28]. These data can then be used to obtain highly detailed (< 1 μ m resolution) 3D segmentations of the different tissues and cell shapes that can be used to reconstruct their 3D shapes. Finally, confocal microscopy has also been used to image calcium dynamics in live mouse embryos at the start of primitive heart tube beating [44], albeit for short periods of time.

Despite its advantages, conventional confocal microscopy is relatively slow and harsh on the sample. The laser illuminates the whole thickness of the sample, scanning point-by-point for extended times, which causes phototoxicity and bleaching [45]. This is a limitation both to image large 3D volumes and to keep embryos alive for long periods. The implementation of light-sheet fluorescence microscopy (LSFM) reduces these problems and expands the ability to image sensitive developing organisms. Using a laser sheet to selectively illuminate the embryo along the detection focal plane, an entire section is captured at once with a camera, significantly reducing phototoxicity [46,47]. This approach has been successful at imaging primitive heart tube and looping stages in both in mouse [17,26,27,29] and zebrafish [46,48] live embryos.

As an alternative, two-photon microscopy employs an excitation laser with twice the wavelength of the desired excitation (such as the one used in conventional single-photon confocal), which reduces light scattering within a sample by up to 1/16 [49]. This method localizes the fluorescence excitation at the focal point of interest, allowing for three-dimensional resolution, similar to that of a conventional confocal laser scanning microscope, while also minimizing phototoxicity on out-of-focus planes and improving penetration, thanks to the longer wavelength. Two-photon systems have been used to image live mouse embryos, resolving primitive heart tube development up to 300 μ m deep in the sample [2]. As confocal, the two-photon is also a point scanning technique, which limits its imaging speed, and thus its application to live imaging of mouse embryos is limited to early-stage whole embryos or selected subregions of bigger embryos.

Thus, when comparing two-photon and light-sheet microscopy for live imaging, two-photon is better for imaging deep into scattering tissues because of its use of near-infrared light and selective excitation. However, its acquisition speed is limited as it builds up the image point-by-point. Light-sheet microscopy offers faster acquisition speed and even lower photodamage, though in exchange for a slightly worse resolution in deep tissue layers in live embryos [50]. Recently, a two-photon light-sheet system has been developed and implemented in the development of zebrafish hearts [51], opening promising avenues for improving live imaging by combining the best features of both approaches.

**Table 1:** A comparison of the different imaging approaches.

	Imaging Approach	Principle	Acquisition Depth (mm)	Advantages	Limitations	Applications	Heart References
**Scanning laser**	Confocal	Pinhole-restricted focal plane with high-power illumination of the whole optical path.	0.5–0.7	High resolution, detection of multiple fluorescent molecules.	Harsh for live sample; high photodamage; high bleaching.	Fixed samples, very high resolution, multiple fluorescent signals. Short time live imaging. Resolves cellular and sub-cellular details.	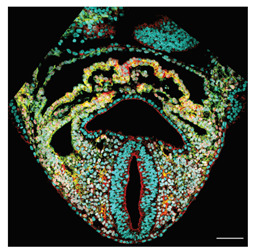 [28] ^**a**^
	2-photon	Point-by-point selective high-power excitation by simultaneous absorption of two low-energy photons in a single event.	1	Deeper penetration and less photodamage than confocal.	Slow, weaker absorption, increased temperature of the sample, broad wavelength excitation bands, possible signal overlapping. Requires bright reporters.	Live imaging of small samples, cell tracking, cell shape, and filopodia analysis. Imaging of fixed scattering samples.	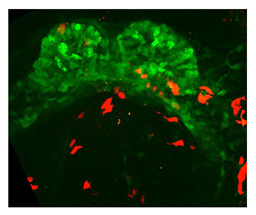 [2] ^**b**^
	Light-sheet	Thin-sheet illumination by a laser light to illuminate a sample from the side, while a camera positioned perpendicular to the light sheet captures images of the illuminated plane at once.	0.5	Fast and minimal photodamage. Cell tracking, cell shape and filopodia analysis.	Needs clarification for non-translucid samples. Live imaging: less definition than two-photon in deep tissue layers due to light scattering.	Quick image of fixed and clarified large embryos. Live imaging of translucid embryos at high temporal resolution.	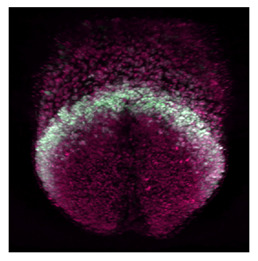 [17] ^**c**^
**Stereology**	Histological Sections	Cutting paraffin embedded or frozen tissue into thin slices.	Whole samples.	Widely available and common, optimal staining.	Variable and unpredictable distortions produced by tissue sectioning and staining.	2D imaging, super-resolution microscopy.	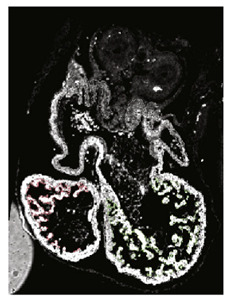 [52] ^**d**^
	HREM	White light reflection on the surface of histology blocks serially sectioned.	12	Histologic quality at high resolution in whole mounted samples.	Weaker contrast on big samples. Sample preparation and imaging are time-consuming. Color reactions (Xgal or BCIP/NBT) required for specific labeling of gene activity.	High-resolution images from 3D structures. 3D cell shape analysis.	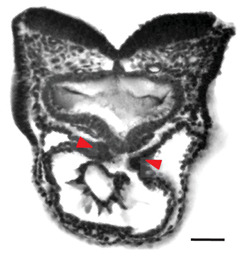 [15] ^**e**^
**Tomography**	Micro-CT	Differential X-ray attenuation depending on tissue density.	500	Resolves thick and opaque tissues.	High radiation dose. Soft tissues need additives for contrast. Not suitable to detect gene/marker expression patterns. Does not reach a cellular resolution.	Big samples and advanced embryos. Isotropic 3D tissue reconstruction.	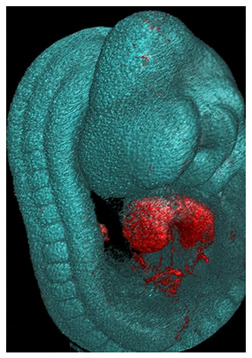 [53] ^**f**^
**Tomography**	OCT	Optical scattering based on changes in its refractive index.	2	Fast and non-invasive. Label-free.	Limited molecular information. Not suitable to detect gene/marker expression patterns or detect individual cells.	Live imaging in utero and ex utero. Isotropic 3D tissue reconstruction.	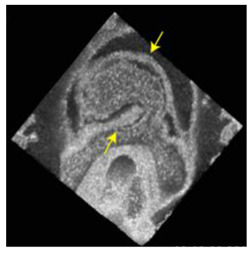 [54] ^**g**^
	OPT	Optical equivalent of micro-CT. It uses ultraviolet, visible, and near-infrared photons instead.	10	Detects specific fluorescent signals, reporters and stains.	Requires sample clarification with an organic solvent that may disrupt antibody stainings.	Resolving specific fluorescent and histochemical reporters. Isotropic 3D tissue reconstruction.	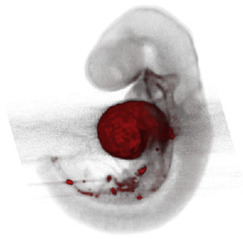 [55] ^**h**^

Note: **^a^**, Frontal optical section of a mouse heart at E8.0. Reproduced with permission from [28]. **^b^**, Frontal maximum intensity projection of a mouse heart at E8.0. Reproduced with permission from [2]. **^c^**, Frontal maximum intensity projection of a whole mouse embryo at E7.5. Reproduced with permission from [17]. **^d^**, Cranial optical section of a mouse heart at E11. Reproduced with permission from [52]. **^e^**, Cranial optical section of a mouse heart at E8.5. Reproduced with permission from [15]. **^f^**, 3D Reconstruction of a whole mouse embryo at E9.5. Reproduced with permission from [53]. **^g^**, Frontal optical section of a mouse heart at E8.5.Reproduced with permission from [54]. **^h^**, 3D reconstruction of a whole mouse embryo at E9.5. Reproduced with permission from [55].

### 2.2. Stereology

The analysis of thicker, whole-mounted samples requires alternative approaches. Among them, tissue stereology is a quantitative method used to estimate the three-dimensional characteristics of an object based on measurements made on two-dimensional sections of the object. Anton Moorman and colleagues have used fluorescence microscopy and histology on tissue sections and stereological methods in the study of heart development in chicken, mice, and human embryos. They have produced atlases that provide a comprehensive overview of the anatomy of the developing heart, together with quantifications of cell proliferation and apoptosis [52,56,57]. These histological images reveal the intricate details of cardiac tissue and cellular structure.

However, 3D images obtained from realigned sections lose some of the tissue architecture information due to variable and unpredictable distortions during tissue sectioning and staining. As an alternative to avoid these problems, episcopic 3D imaging is a technique that involves using a series of consecutive digital images obtained from the block surface of methacrylate resin-embedded tissues or embryos. These images are captured during the physical sectioning of the biological sample, which results in a complete, aligned, and undeformed dataset. images are obtained directly from the face of the resin block, rather than glass-mounted sections; the 3D reconstruction is inherently complete and free from errors and distortions that can arise during sectioning and mounting [58,59]. High-resolution episcopic microscopy (HREM) is particularly useful for exploring the changing morphology of the developing heart because it provides accurate data sets, resolving down to 1 μ m details. This enables accurate measurement of individual structures, allowing for a more systematic and quantitative analysis of changes in heart structure and composition during embryonic development. Such imaging methods provide an objective baseline for identifying developmental abnormalities that may be difficult to assess by qualitative criteria alone, such as the unknown normal range of variation in ventricular trabeculation and mild phenotypes that could be the basis of non-compaction disease [60].

### 2.3. Tomography

Although HREM is a valuable imaging technique to resolve 3D details on internal structures of large samples, it can be time-demanding for large samples. Micro-computed tomography (micro-CT) offers an alternative to obtaining this type of data. This technique is based on the measurement of differential X-ray attenuation of tissues related to their density. For example, iodine contrast micro-CT produces detailed imaging of intact embryos without causing physical damage, with a resolution ranging 3 μ m to 11 μ m [20]. Detection of cardiac phenotypes by micro-CT is suitable especially for late embryos due to the internalization of the heart in later stages [20]. For example, *CFAP300* mutations have been associated with pre-ciliary dyskinesia using this imaging approach [61].

With similar reconstruction algorithms, 3D optical projection tomography (OPT), an optical type of tomography, was used to reconstruct the growth of the looped mouse heart. Scans of mouse embryos were manually aligned and processed to create surfaces that were then characterized and interpolated over the developmental stages to reconstruct the heart trajectory [55].

Recently, optical coherence tomography (OCT) has also been applied in live imaging of mammalian development. Larina et al. [62] showed that OCT can be used to image embryos developing in utero in a noninvasive way. Lopez and Larina [63] adapted this methodology to image earlier embryos cultured ex vivo, improving the resolution to 1 μ m. These studies show that, when cell-segmentation or tracking is not required, OCT offers a fast tool for quantifying heart tube morphogenesis and hemodynamics in live embryos [62,63,64]. Overall, OCT is a label-free imaging method with several advantages for imaging cardiac development in fixed and live samples. It has a high spatial resolution ( 2 μ m–20 μ m), which enables visualization and measurement of cardiac layers and fine structures [65]. Its high temporal resolution of 10 to >100 kHz line rates allows for assessment of cardiac dynamics also in three dimensions [66]. With an appropriate imaging depth of 2 mm–2 mm, OCT allows imaging of four-chamber heart development under physiological conditions [67,68]. Finally, Doppler OCT enables measurement of hemodynamics and biomechanical forces induced by blood flow, while vascular mapping enables visualization and measurement of the vasculature [69].

## 3. Quantitative Approaches for Heart Morphogenesis

In this section, we review the quantitative strategies used to study heart morphogenesis. Specifically, we focus on mathematical approaches implemented to quantify heart morphogenesis at the tissue and cellular level, using 3D (static) and 3D+time (live) images (see Table 2, Table 3 and Table 4). These quantitative analyses are constrained by the datasets available, which depend on the imaging approach used to obtain the data.

### 3.1. Static Imaging

#### 3.1.1. Tissue-Level Analysis

When combined with clarification methods, imaging fixed samples generally offers higher resolution than imaging live embryos. Static imaging captures snapshots of tissues and cells during development, providing multiscale information to infer morphological characteristics. At the cellular level, it allows for the extraction of morphometric and morpho-mechanical features from nuclei and membrane segmentation.

When studying heart morphogenesis at the tissue level, researchers have primarily used quantitative approaches that involve extracting geometric parameters from three-dimensional (3D) reconstructions to identify key morphometric changes. One of these studies, conducted by Le Garrec et al. [15], explored the mechanical causes behind heart looping by measuring simple geometric parameters from 2D sections extracted from HREM 3D reconstructions. They measured heart tube elongation by measuring the length of a line passing through the centroids of all cross-sections. They also defined the timing of the breakdown of the dorsal mesocardium by measuring its change in thickness along the arterial to venous poles at different stages. Finally, they quantified the right and left angles between the heart tube and the dorsal pericardial wall, demonstrating a left-right asymmetry observable at the poles.

In a more recent study, Esteban et al. [28] examined 3D reconstructions of the myocardium and surrounding tissues of more than 50 specimens from cardiac crescent to heart looping (E7.75–E8.5), developed a new morphometric staging system, and registered the specimens in time and space, generating a pseudo-dynamic Atlas. The Atlas describes the average morphological evolution of the specimens and provides a map of regional morphological variability at each stage. Taking advantage of the Atlas, they computed the evolution of the angle formed by the inflow tracts and the orthogonal embryonic axes, revealing an early and progressive divergence between the angles on the left and right side (around E8.0), which constitutes the first left-right asymmetry described in the heart. Furthermore, they found that this asymmetry was reversed in *Nodal* mutants, in which heart looping in randomized, identifying a genetic base for this asymmetry and its causal relationship to looping.

Unlike the studies mentioned above, Paun et al. [70] implemented an innovative framework to compare the hearts of different specimens even before extracting geometric parameters. They used alignment and parametrization techniques to quantify trabecular morphogenesis of the heart ventricle in the mouse embryo from E14.5 to E18.5. For the first time, they quantified trabeculation using 3D fractal analysis on the volume and surface area of ventricle reconstructions from HREM images. To standardize the analysis and make it independent of geometry, they parameterized the entire surface area of the ventricles on a planar domain by flattening the tissue in their longitudinal direction as a cartography. The results showed an increased complexity of myocardium tissue from the bottom to the top of the ventricle and a decrease in overall complexity with advancing gestational age. This framework offers new possibilities for examining abnormal trabeculae in cases of congenital heart disease. In this case, 3D analysis was essential, given that, indeed, two-dimensional (2D) images alone did not provide easily comparable data due to variations in the trabeculae’s geometry.

In general, the use of quantitative approaches based on 3D reconstructions of the forming heart at tissue resolution has provided important insights. These studies have revealed conserved features of the heart morphology that precede key events in heart morphogenesis and established frameworks to detect early phenotypes in disease models.

#### 3.1.2. Cellular-Level Analysis

Analyzing the cellular level of the heart tube plays a critical role in understanding the factors behind its formation. Characteristics of cell shape, size and orientation provide insights into the magnitude, regionalization and anisotropy of tissue growth and the mechanical forces involved in heart morphogenesis. Many quantitative approaches have been implemented to analyze cardiac morphogenesis at the cellular level, involving the extraction of geometric parameters.

Le Garrec et al. [71] developed a method to examine tissue anisotropy at the cellular level using 3D confocal imaging of the mouse heart. They used image segmentation, statistical analysis, and clustering techniques to determine the local coordination of cell polarity and division orientation in the embryonic mouse heart. They found that there was a significant local anisotropic coordination of ventricular cardiomyocytes at early embryonic stages. As a limitation, this study did not address the 3D nature of the problem, using flat projections for the analysis and focusing on subregions of the forming ventricles and not the whole structure.

Francou et al. [72] also studied cell anisotropy but focused their research on the behavior of the dorsal pericardial wall (DPW) cells in the mouse embryo. This region contains cardiac progenitors of the second heart field, which would be added to elongate the heart tube in subsequent stages. They used membrane markers to perform a thorough quantitative analysis at the cellular level based on shape geometry. They used this information to estimate the mechanical forces that cells experience and deduce anisotropic tension and cell stretching during tissue morphogenesis. They observed a polarized mechanical stress acting on cells in the posterior part and that the direction of cell elongation was significantly less oriented toward the arterial pole. They also used unsupervised classification of cell shapes to define morphological cell clusters, which distribution showed biased and directional behavior, oriented towards the arterial pole.

Along these lines, but focusing on the differentiation of the first heart field precursors, Ivanovitch et al. [2] measured the geometric parameters of the cell shapes to assess the changes associated with cardiomyocyte differentiation. They evaluated the roundness of cells from 2D images to characterize the difference between cardiac progenitors and differentiated cardiomyocytes, revealing that the latter was rounder and had lost columnar epithelial organization. Furthermore, they identified a transit zone in which the differentiating cardiomyocytes (according to marker expression) did not show yet a round shape. This observation suggested that changes in cell morphology alone were insufficient to distinguish differentiated cardiomyocytes, and detachment from the endoderm was also required.

While the previous studies focused on localized parts of the developing heart, Ebrahimi et al. [73] proposed a novel quantitative method to study cell shape at the whole heart. This was achieved by developing a computational method to measure morphometric parameters at the cell level during the C-looping stage (HH10–HH11, chicken). They combined 3D confocal microscopy and micro-CT imaging techniques to create a multi-scale 3D dataset of the entire chick heart using the finite element method. They used OpenCMISS software [74] to fit an initial bicubic-linear tubular mesh to the specific anatomical points of the heart for each sample, aligning them in space and time using rigid registration based on anatomical landmarks. Morphometric features were extracted directly from cells by segmenting labeled myocardial cells from 3D confocal image stacks using a deep learning base model. Different cell properties such as volume, anisotropy, and orientation were quantified and mapped to the surface of the corresponding finite element geometry of the given sample. The features were compared, showing a region-based differential spatiotemporal pattern for cellular features spatially and temporally. Although limited in conclusions due to the low number of specimens analyzed, this study represents a good example of how to integrate spatial information at different scales in the context of the whole developing organ to extract local quantitative features.

Another important contribution to the incorporation of cellular parameters to whole-organ descriptions was achieved by de Boer et al. [52], who generated stereological atlases of several stages of the developing mouse heart and surrounding tissues. They created the first 3D atlas of the myocardium and the surrounding splanchnic mesoderm ranging from the early cardiac crescent to the prenatal four-chambered heart. To quantify proliferation, they measured the proportion of BrdU-positive cells and mapped these data on 3D structures, revealing proliferation patterns that may drive morphogenesis. Along these lines, Meilhac et al. [4] had previously used data from retrospective clonal analysis to describe a regionalized pattern of proliferation in the early heart tube, however, did not provide a 3D study of this feature.

### 3.2. Live Imaging

Live imaging provides temporal information, allowing analysis of spatiotemporal morphogenetic events at both the tissue and the cellular levels. Tissue-level analysis using live imaging can track tissue movement to reveal the kinetics and deformation of heart tissues. In vivo imaging at the cellular level allows tracking of cell migration, division, and rearrangements, following the fate of individual cells as they differentiate into specific cell types. Fluorescence-based reporters enable the monitoring of specific gene activity in real time, providing information on spatial and temporal patterns of gene expression during the development.

#### 3.2.1. Tissue-Level Analysis

Live imaging is a powerful tool for investigating the spatial and temporal dynamics of heart development. As such, efforts have been dedicated to developing advanced tools to extract tissue-level information from live imaging data.

One of the problems in conceptualizing the results obtained from the 3D live analysis is the complexity of 3D shape evolution. In this context, Heemskerk et al. [75] proposed a framework called ImSAnE (Image Surface Analysis Environment) for automatically constructing an atlas of 2D maps for dynamic tissue surfaces. ImSAnE isolates a 3D folded surface and transforms it into a sequence of 2D images that are superimposed on each other like an Earth map. Heemskerk et al. applied ImSAnE to light-sheet images of the beating heart of zebrafish, showing that the framework can recreate the cartography of the 3D heart and minimize map distortions by parametrization. By mapping the heart to a plane, they were able to follow cells in a heartbeat with relative ease and with less computational infrastructure, saving time and resources.

A further important problem when analyzing heart development data is dealing with the heartbeat. Lee et al. [76] designed a strategy for eliminating heartbeat artifacts in zebrafish live images for studying the trabeculation process. The team revealed the relationship between trabeculation and the contractile ability of the ventricle by analyzing synchronized 4D SPIM images of the zebrafish heart. They performed the analysis in normal embryos, in mutant embryos with reduced atrial contractions, and in embryos with reduced hemodynamic force. To understand the interaction between myocardial contractility, hemodynamic shear stress, and trabecular formation, they correlated changes in ventricular strain, fractional shortening, and ejection fraction with the trabecular structure. To assess ventricular strain, they calculated changes in circumferential displacement (D). To determine D changes, they segmented the ventricular wall and generated a boundary mesh. The mesh nodes were then adjusted to follow the motion of the ventricular wall captured by SPIM. The fractional shortening was defined as the difference between the end-diastolic displacement and end-systolic displacement. Additionally, the ejection fraction was calculated by comparing the end-diastolic and end-systolic volumes of the ventricle, calculated in the 3D reconstruction with Amira software [77]. To evaluate the trabecular structure, they assessed the changes in the volume of the trabecular myocardial ridges. This was done by subtracting the volume of the smooth curves, which did not include the trabecular ridges, from the overall myocardial volume. Their quantitative approach revealed a close relationship between trabeculation, contractility, and shear stress. This study is therefore a nice example of accounting for heart physiology while investigating its morphogenesis in live analysis.

Regarding heart looping in the chicken embryo, Kawahira et al. [78] proposed a quantitative approach to map the deformation of the myocardium during C-looping. They collected 3D+t images using a two-photon microscope. To reconstruct a deformation map of the looping process, they applied a Bayesian method on 2D cell coordinates using spherical harmonic expansion. By applying solid mechanic laws, they extracted the growth rate and anisotropy deformation for the left and right sides of the heart. The growth rate was defined as the variation of the mesh area, the anisotropy as the ratio between the principal eigenvalues and the deformation direction as the eigenvector with maximum eigenvalue. Their dynamic analysis revealed that the growth rate was similar between the two sides, suggesting that differential growth does not play a significant role in C-looping. However, deformation anisotropy showed a left-right asymmetry: while the left side underwent circumferential stretching, the right side exhibited longitudinal elongation. Therefore, this study provided a nice example of how the polarity of tissue deformation contributes to heart tube morphogenesis.

#### 3.2.2. Cellular-Level Analysis

To understand how tissue-level transformation emerges from cell behavior, information about dynamic changes in cell shape and organization is crucial. Live imaging approaches have been employed in several studies to characterize variations in cell shape, displacement, rearrangement, and migration and how these could explain cardiac tube shaping.

In the study by [78], authors aimed to understand the cellular basis of differential L-R tissue anisotropy. To achieve this, cells were segmented and various geometric parameters such as volume, area, orientation, and anisotropy were determined from cell shape. Cellular features were then approximated by applying them to a 3D ellipsoid and compared between the left and right sides; however, the individual cell parameters did not explain the tissue-level anisotropy. The authors then focused on cellular rearrangement. They captured 3D time-lapse images of tissue patches at single-cell resolution from the left and right sides every 10 min and tracked approximately 30 nuclei per patch. For tracking, they used a tensor decomposition method that measures cell rearrangement as the difference between two kinds of tensors: the tissue velocity gradient tensor (LT) and the cell shape strain rate tensor (LC). LT represents the deformation per unit of time of a cell population, whereas LC represents the change in cell shape. Their study revealed, for the first time, that a dynamic rearrangement of cells within the tube plays a substantial role in left-right asymmetric tissue deformation during C-looping and is a nice example of how quantitative tracking of cell rearrangement informs the causes of tissue deformation.

Using live imaging of in vitro cultured mouse embryos, Ivanovitch et al. [2] used a random genetic cell lineage labeling to trace tissue and cell displacement and map the differentiation schedule of first and second heart field precursors. They described sliding of the splanchnic mesoderm over the ectoderm as an essential morphogenetic movement for transforming the cardiac crescent into the heart tube and identified alternating phases of differentiation and morphogenesis during this process. This study allowed the temporal calibration of these events with an estimated speed of the splanchnic mesoderm moving towards the midline between 12 and 20 μ m/ h ^−1^. They concluded that it takes approximately 5–7 h from the late cardiac crescent stage to the open HT stage for the splanchnic mesoderm to reach the midline.

Similarly, Francou et al. [79] used explanted mouse tissues for the live imaging of the apicobasal polarization and basal cell dynamics to characterize the properties of SHF in the dorsal pericardial wall. They analyzed the apicobasal polarization and cell morphology between E8.5 and E9.5 by determining the circularity index and measuring the length and lifetime of the filopodia located on the basal membrane of the cells. Analyzing a *Tbx1* mutant, which affects SHF progenitor addition to the heart tube, they revealed increased circularity and reduced filopodial activity in those same progenitors. Their findings highlight the importance of the epithelial properties of SHF progenitor cells in promoting heart development.

While the studies above have characterized only local cell displacements or traced reduced numbers of labeled cells, describing the complexity of cellular contributions to tissue morphogenesis requires the global tracing of all individual cells in the field of interest. Towards this goal, improvements in spatial and temporal resolution of live imaging have led to the extraction of individual cell behaviors, which involves the development of sophisticated computational tools for automatically tracking cellular movement [80]. These tools typically involve two parts: initially, an object detection algorithm segments individual objects in each frame; then, a linking mechanism connects these objects to create tracks. Currently, state-of-the-art segmentation techniques make use of advanced machine learning and deep learning methodologies.

McDole et al. [26] introduced the revised version of the Bayesian cell tracking framework, known as TGMM 2.0. The algorithm achieved an accuracy rate of 99.8% when tested on 381 manually curated tracks. To improve tracking on long-term experiments, the authors combined TGMM 2.0 with statistical vector flow analysis, providing a comprehensive understanding of cell trajectories as cells moved from the primitive streak to their specific mesodermal region. They tested the framework in 4 mouse embryos and, for the first time, created a 4D atlas of the entire embryonic morphogenesis process from gastrulation to early organogenesis (E6.5–E.8.5). Although this method excels at tracking flows of groups of cells, it was not designed to track individual cell identities over long periods of time, leaving a challenge for future tracking algorithms.

In this context, Yue et al. [27] proposed a new pipeline, called GrapeBio, which coupled with the TGMM tracking algorithm generates continuous cell lines of the developing mouse heart from E8.25–E9.5. Their findings revealed new cellular mechanisms that drive the ballooning and trabeculation process, as both cell intercalation and horizontal cell division were found to be contributing factors in these processes.

### 3.3. Computational Modeling

Biological systems are complex structures with a hierarchy of components organized into sub-cellular, cellular, tissue, organ, and organism levels. Consequently, alterations in any of the components may influence the others. Cellular mechanisms control and regulate the forces that define the structure of the tissue, while the structure of the tissue forces the cells into a given position, affecting the signals that the cells exchange and thus conditioning their behavior. This is why understanding an organ’s development requires characterizing the various components and exploring the intricate relationships between these at different scales. Mathematical and computational modeling approaches provide the possibility of integrating all this information to test the influence of individual components.

For example, in heart development, mathematical and computational modeling have been used in parallel with experimental methods to simulate morphogenetic events such as heart looping. Some of these models establish a determined direction of the loop [81], while others investigated the bending process and did not address the more complex mechanism of C-looping. Shi et al. [82] used a computational model to assess the hypothesis that heart bending during looping is due to differential hypertrophic growth [83]. Simulating the bending of the heart as a continuous sheet, they noted not only differential growth but also other factors that contributed to the bending of the heart, including change in active cell shape change [84,85], cardiac jelly swelling, dorsal mesocardium tension [86] and myocardial contraction [87], as described in previous experimental studies.

Meilhac’s group [15] proposed a finite element model to simulate the C-looping process and test their experimental observations. They treated the heart tube as a continuous sheet of material in the form of a regular cylinder and found that C-looping involved a buckling mechanism, resulting from specific mechanical constraints and left-right asymmetries, together with heart tube elongation. In their study, they initially simulated a differential tissue expansion in the circumferential direction. The contraction of the right side caused the arterial pole to rotate to the right. Later on, differential growth at the poles in the longitudinal direction, combined with the gradual breakdown from the dorsal mesocardium, resulted in bending in opposing directions, ultimately giving rise to a helical form.

Computational modeling is a useful tool for studying relevant processes in congenital heart diseases. Desgrange et al. [88] used simulations to investigate left-right asymmetry in the hearts of *Nodal* mutant mice. They found that *Nodal* is not necessary for heart looping initiation but instead modulates asymmetries at the poles of the heart tube. The researchers identified four classes of anomalies characterized by reduced asymmetries at the poles in terms of variable laterality and intensity. Simulations of a 50% reduction in intensity were sufficient to reproduce the mutant shapes, as defined by the position of the ventricles. The model was further validated by predicting the orientation of the right ventricle-left ventricle axis in all classes of shapes, which showed a strong correlation with the biological values. Therefore, the model, which included randomized laterality and a reduction of asymmetries at the poles, was able to recapitulate *Nodal* mutant shapes. These findings demonstrate that *Nodal* is necessary for amplifying and coordinating left-right asymmetries at the poles of the heart tube to generate a robust helical shape.

Recently, Honda et al. [89,90] used a computational simulation based on the cell-vertex model to verify looping conditions at the cell level in an idealized heart tube model. The cell-vertex model not only introduced the shape of the tissue but also allowed the setting of individual cell properties, controlling the shape, migration, division, and intercalation of cells during the morphogenetic process. In their model, the primitive heart was approximated as a straight tube model in which individual cells were assumed to be polygons without thickness. Since tissue bending has been shown in the literature to be due to the differential proliferation of cardiomyocytes, Honda et al. performed a computer simulation assuming that the cells on the ventral side were in a state of proliferation. The simulation showed that not only differential growth but also directional growth is required for bending. In fact, the cells were set to divide along the longitudinal axis of the heart tube. Under these simulated conditions, the heart did bend. The differential and directional growth, however, did not explain how the heart obtained its helical structure.

To explain the left-right asymmetry that generates the helical shape, researchers then applied rotational and frontal displacement to the anterior region of the tube to explain the left-right asymmetry that generates the helical shape. The results indicated that the displacement of the tube causes the left-handed screw formation of the loop. An experiment was proposed at the cellular level, and the computational model suggested that the rotational deformation of the tissue influenced the behavior of individual dividing cells.

In addition to the above models, which recreate heart tube morphogenesis without accounting for its function, computational models have been used to numerically model hemodynamics and how this affects cardiac morphogenesis [91,92,93,94,95]. Numerical modeling offers a wider field for spatiotemporal investigations, allowing the determination of hemodynamic parameters in the entire flow domain during heart development. One of the most widely used numerical approaches is computational fluid dynamics (CFD) modeling, which solves fluid dynamic equations using realistic image-based cardiac geometries. Salman [96] provides a comprehensive review of the various uses of computational modeling to study the haemodynamics of blood flow in the heart.

Here, we would like to highlight a specific application that uses 4D CFD modeling together with whole-organ imaging to quantitatively analyze the mechanobiological impact of hemodynamics on heart trabeculation [97]. In their study, Vedula et al. [97] proposed a framework that combines light-sheet imaging and CFD simulation to quantify the biomechanical forces involved in cardiac trabeculation in terms of endocardial wall shear stress (WSS) and oscillatory shear index (OSI) from the velocity field provided by the CFD simulation. Results showed higher levels of WSS in healthy hearts than in treated hearts with trabecular defects. Higher levels of OSI were also found in healthy hearts in the trabeculae grooves than in the ridges, indicating that oscillatory forces may be a potential mechanism for regulating the development of cardiac trabeculation.

**Table 2:** Quantification approaches at **tissue-level**.

	Reference	Biological Insight	Image Approach	Quantitative Approach	Measurement	Stage
**Tissue-Level**	**Esteban et al.** (2022) [28]	A continuous 4D digital Atlas of heart development. First of L-R asymmetry in mouse heart detected in the IFTs directions.	3D confocal images.	3D mesh of the heart tissues. Morphometric staging system.	IFT directions defined as the angles between the IFT axis and craniocaudal embryo plane at different stages.	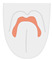 _a_
	**Ivanovitch et al.** (2017) [2]	Different growth rates and splanchnic mesoderm migration shape the mouse heart: 1.earlyCC-to-lateCC growth rate ↑; 2.CC-to-openHT growth rate ↓; 3.openHT-to-linearHT growth rate ↑.	3D confocal images.	3D tissue segmentation.	Volume estimation at different stages.	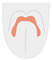 _a_
	**Le Garrec et al.** (2017) [15]	Sequence of the main mechanical events driving looping in mouse heart: ^1^ HT elongation; ^2^ progressive breakdown of the DM; ^3^ L-R asymmetry at the poles.	HREM images; 2-photons 3D+t images.	Mesh reconstruction of planar section.	^1^ Axis length intersecting the centroids of each 2D reconstruction; ^2^ DM thikness in different planar section over time; ^3^ L-R angles between HT and DM.	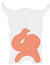 _b_
	**Kawahira et al.** (2020) [78]	^1^ Tissue motion map of the chick heart during looping C shows an L-R asymmetry in the ^2^ direction of deformation. Left side with circumferential stretching, right side with longitudinal elongation. Comparable ^3^ growth rate between L-R.	2-photons 3D+t images.	^1^ Cellular tracking + SHE + Bayesian method resulted in 3D+t tissue mesh. ^2–3^ Continuum Mechanics laws on the tissue mesh.	^2^ Anisotropy deformation and deformation direction as the main eigenvalue. ^3^ Growth rate.	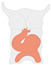 _b_
	**Yue et al.** (2020) [27]	Cell intercalation and directional proliferation are driving forces in ballooning and trabeculation process in mouse hearts. Cellular intercalation(+) and horizontal division(++) drive ballooning process. Early cell fate(+), oriented cell division and directional migration(++) drive trabeculation process.	3D+t vLSFM images.	3D nuclei segmentation, nuclei tracking.	Angle between the parent-to-daughter line and the line connecting the centroid of the left ventricle and the parent cell.	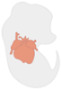 _c_
**Tissue-Level**	**Le Garrec et al.** (2013) [71]	^1^ Cell polarity and ^2^ oriented cell division drive planar expansion of the mouse ventricle with ^3^ local coordination.	3D confocal images.	3D cellular segmentation, statistical test, clustering algorithm.	^1^ Axis nucleus-the centrosome. ^2^ Orientation of cell division as the angle between the two daughter nucleus. ^3^ spatial correlation function.	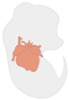 _c_

Note: Heart morphogenesis stages related to Figure 1. _a_, Early heart formation in a mouse embryo. _b_, Lengthening and looping of the mouse heart. _c_, Formation of the chambered heart in a mouse embryo.

**Table 3:** Quantification approaches at **cellular-level**.

	Reference	Biological Insight	Image Approach	Quantitative Approach	Measurement	Stage
**Cellular-Level**	**Dominguez et al.** (2023) [17]	Spatiotemporal organization of cardiac cells in mouse model.	3D+t LSFM images.	Nuclei tracking.	Nuclei orientation, spatio temporal displacement of cells in FHF, SHF and juxta-cardiac field.	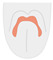 _a_
	**Ivanovitch et al**. (2017) [2]	^1^ Differentiation schedule tracing showed alternating phases of differentiation and morphogenesis during heart tube formation; ^2^ Timing early morphogenesis events in a mouse heart. CC-to-openHT in aprox 5–7 h.	^1^ 3D confocal images. ^2^ 2-photons 3D+t images.	^1^ Cellular segmentation in 2D section. ^2^ Cellular tracking.	^1^ Roundness index. ^2^ Cell speed at the border to reach the embryo midline.	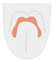 _a_
	**de Boer et al.** (2012) [52]	Proliferation pattern in mouse HT: Asymmetric L-R ventral myocardium growth. High proliferation in SPL.	Fluorescence images.	3D reconstruction+ design-based stereological method.	Proliferation rate (fraction of proliferating cells).	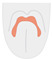 _a_
	**Ebrahimi et al.** (2022) [73]	Cellular changes during C-looping reveal spatiotemporal patterns of differentiated growth in chick hearts, highlighting the inter-cellular space’s relevance.	3D confocal images; μ mCT.	3D cell segmentation + 3D tissue mesh.	Geometric parameters (cell volume, cell density, cell anisotropy).	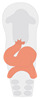 _b’_
**Cellular-Level**	**Kawahira et al.** (2020) [78]	Right-specific directional cell rearrangement during looping in chick heart. No significant differential growth between L-R sides.	2-photons 3D+t patch images.	^1^ Nuclei tracking; ^2^ tectonic theory from Blanchard et al.; ^3^ 3D cell segmentation and ellipsoid approximation.	^2^ Cell rearrangement is defined as the difference of tissue and ^1^ velocity gradient tensor and the ^3^ cells shape strain rate tensor; ^3^ geometric parameters (cell volume, cell orientation, cell anisotropy, cell section area).	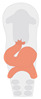 _b’_
	**Francou et al.** (2017) [79]	Epithelia tension promotes mouse HT elongation.	2D confocal images.	Cellular segmentation.	Geometric parameters(cell elongation, cell orientation). Orientation of cell division as the angle between the two daughter nuclei.	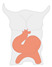 _b_
	**Francou et al.** (2014) [72]	^1^ Cell polarity and ^2^ filopodia activity in SHF play an important role in mouse HT elongation and OFT morphogenesis.	^1^ 2D confocal images; ^2^ 2-photons of thick-slice tissue.	Cellular segmentation.	^1^ Circularity index, apical/basolateral membrane ratio. ^2^ Filopodia max length and filopodia lifetime.	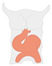 _b_
**Cellular-Level**	**de Boer et al.** (2012) [52]	Proliferation pattern in mouse HT: asymmetric ventrodorsal myocardium growth. High proliferation in the outer curvature. Low proliferation in the inner curvature. High proliferation in SPL.	Florescence images.	Design-based stereological method; 3D reconstruction.	Proliferation rate (fraction of proliferating cells).	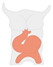 _b_
	**Paun et al.** (2017) [70]	Spatiotemporal characterization of the complexity of myocardium tissue during mouse heart trabeculation. The complexity of trabeculations increases longitudinally from the base to the apex during gestational age.	HREM images.	3D reconstruction; geometry independent representation to establish correspondence between different objects.	3D fractal dimension, myocardia volume, myocardial surface area and the ratio between the two.	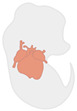 _c_
	**Lee et al.** (2016) [76]	Close link between ^1^ trabeculation and ^2^ contractile ability of the ventricle in zebrafish model.	3D+t SPIM images.	Synchronization algorithm for heart beating. 3D segmentation + 3D tissue mesh.	^1^ Trabecular structure(volume changes of the trabecular myocardial ridges). ^2^ Ventricular strain (changes in circumferential displacement), fractional shortening(difference between the end -diastolic displacement and end-systolic) and ejection fraction(end-diastolic on end-systolic volumes of the ventricle).	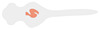 _c’’’_
**Cellular-Level**	**de Boer et al**. (2012) [52]	Proliferation pattern in mouse heart: low proliferation in the OFT. High chambers proliferation.	Florescence images.	Design-based stereological method; 3D reconstruction.	Proliferation rate (fraction of proliferating cells).	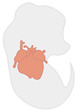 _c_

Note: Heart morphogenesis stages related to Figure 1. _a_, Early heart formation in the mouse embryo. _b_, Lengthening and looping of the mouse heart. _c_, Formation of the chambered heart in the mouse embryo. _b’_, Formation of the chambered heart in the chick embryo. _c’’’_, Formation of the chambered heart in zebrafish embryo.

**Table 4:** Computational modeling approaches.

	Reference	Biological Insight	Acting Force	Result	Stage
**Computational Modeling**	**Honda et al.** (2021) [89]	Cell-based modeling combines ventral bending and rightward displacement of the mouse HT.	^1^ Differential proliferation and directional division. ^2^ Anisotropic contractile force of cell edges.	^1^ HT extension and HT bending. ^2^Convergent extension of collective cells	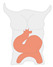 _b_
	**Le Garrec et al.** (2017) [15]	Tissue-based modeling to show that asymmetries at the fixed heart poles, generating opposite deformations, associated with the progressive release of the heart tube dorsally, are sufficient to generate looping of a tube growing between fixed poles.	Boundary constraints, anterior-rightward-biased contraction force.	HT extension, HT bending and left-right asymmetries at the poles	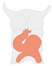 _b_
	**Vedula et al**. (2017) [97]	Computational fluid dynamics modeling to quantify the biomechanical forces involved in cardiac trabeculation in zebrafish model.	Hemodynamic from the blood flow.	Oscillatory forces as a potential mechanism for regulating the development of cardiac trabeculation.	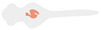 _c’’’_

Note: Heart morphogenesis stages related to Figure 1. _b_, Lengthening and looping of the mouse heart. _c’’’_, Formation of the chambered heart in zebrafish embryo.

## 4. Conclusions and Future Directions

The study of heart development has benefited from advances in imaging technologies and quantitative analysis. The studies discussed here show that confocal microscopy, although relatively slow and harsh on samples, is a powerful tool to obtain highly resolved images in fixed and clarified embryos. When combined with fluorescent staining, it can reveal the 3D location of molecules that play a key role during cardiac development [29,30]. To image live embryos as they develop, light-sheet microscopy provides a faster and less damaging method for imaging the primitive heart tube and looping stages. Two-photon microscopy is a suitable alternative for imaging deep into scattering tissues, although it has limitations in acquisition speed. The combination of two-photon and light-sheet microscopy may offer the best features of both approaches [51].

Thicker, whole-mounted samples are often difficult to resolve with fluorescence microscopy. Other approaches resolve deep into the sample but are often not compatible with fluorescent staining (Table 1). High-resolution episcopic microscopy (HREM) has allowed more systematic imaging of changes in heart structure and composition during later stages of heart development [15], while micro-computed tomography (Micro-CT) offers an alternative to HREM for larger samples, and optical coherence tomography (OCT) is a fast, label-free tool for quantifying heart tube morphogenesis even for embryos in utero. New approaches now allow us to measure the mechanical properties of developing embryos. Brillouin microscopy has been used to image mechanical properties during neural tube closure in live chicken embryos without disrupting its development [98]. This is a promising technique to study the mechanical forces involved in heart development [11].

Each of these imaging methods has some limitations either in terms of imaging speed, spatial resolution, light exposure, or imaging depth. Recently, deep learning algorithms have opened new possibilities for increasing the quality of images after acquisition [35]. With the use of U-nets for image restoration tasks, such as denoising, surface projection or recovery of the isotropic resolution, one can reduce imaging time and laser power without compromising the quality of the obtained images. This is critical for imaging live embryos since phototoxicity and imaging speed play a key role.

Using these raw images to quantify biological phenomena is the first step in understanding how heart development occurs. However, to fully comprehend morphogenesis, it is crucial to identify the origins of the forces that shape tissues during development, as well as their distribution over time and space. Models with appropriate mathematical rigor allow the analysis of heterogeneous experimental data and integrate them across multiple scales without losing biological relevance [15]. These models can predict emergent outcomes, and test biological hypotheses while examining different mechanisms that control a biological system. By using an iterative approach, the modeling output can inform experiments and identify new hypotheses to fill gaps in the experimental data. The recent advances ensure that cooperation and feedback between imaging, quantitative analysis, mathematical modeling and experimentation will provide important insights into understanding cardiac morphogenesis in the near future.

## Data Availability

Not applicable.

## Abbreviations

The following abbreviations are used in this manuscript:

CC	cardiac crescent
CFD	computational fluid dynamics
CS	Carnegie stages
DM	dorsal mesoderm
DPW	dorsal pericardial wall
FHF	first heart field
HH	Hamburger-Hamilton
HPF	hours post fertilization
HREM	high-resolution episcopic microscopy
HT	heart tube
IFT	inflow tract
LSFM	light-sheet fluorescence microscopy
micro-CT	micro-computed tomography
OCT	optical coherence tomography
OFT	outflow tract
OPT	optical projection tomography
OSI	oscillatory shear index
SHE	spherical harmonics expansion
SHF	second heart field
SPIM	selective-plane illumination microscopy
SPL	splanchnic mesoderm
WSS	wall shear stress
