# Correction: Raiola et al. Imaging Approaches and the Quantitative Analysis of Heart Development. *J. Cardiovasc. Dev. Dis.* 2023, *10*, 145

**DOI:** 10.3390/jcdd11070197

**Published:** 2024-06-28

**Authors:** Morena Raiola, Miquel Sendra, Miguel Torres

**Affiliations:** 1 Cardiovascular Regeneration Program, Centro Nacional de Investigaciones Cardiovasculares (CNIC), 28029 Madrid, Spain; morena.raiola@cnic.es (M.R.); msendra@cnic.es (M.S.); 2 Departamento de Ingeniería Biomedica, ETSI de Telecomunicaciones, Universidad Politécnica de Madrid, 28040 Madrid, Spain; 3 Centro de Investigación Biomédica en Red de Enfermedades Cardiovasculares (CIBERCV), 28029 Madrid, Spain

In the published publication [1], there was an error in the affiliation list for Miguel Torres. In addition to affiliation 1, the updated affiliation should include affiliation 3: Centro de Investigación Biomédica en Red de Enfermedades Cardiovasculares (CIBERCV), 28029 Madrid, Spain. The authors state that the scientific conclusions are unaffected. This correction was approved by the Academic Editor. The original publication was also updated.
